# Evaluation of the InTempo path set for CyberKnife prostate and lung SBRT: A single‐institution experience

**DOI:** 10.1002/acm2.70509

**Published:** 2026-02-17

**Authors:** Yingcui Jia, Lei Fu, Shari Rudoler, Eric Gressen, Yevgeniy Vinogradskiy, Qianyi Xu

**Affiliations:** ^1^ Department of Radiation Oncology Thomas Jefferson University Philadelphia Pennsylvania USA

**Keywords:** cyberKnife InTempo path set, lung SBRT, prostate SBRT

## Abstract

**Purpose:**

The InTempo adaptive imaging system is an important component of the Accuray CyberKnife System, designed to enhance the system's ability to track and correct tumor motion during treatment. However, a limitation of this feature is the reduction of available nodes for treatment planning. The impact of a reduced number of nodes on the quality of InTempo‐based treatment plans has not previously been evaluated. This retrospective study aims to compare the dosimetry of CyberKnife plans with and without The InTempo path set for both prostate and lung stereotactic body radiotherapy (SBRT).

**Methods:**

This study included twelve consecutive prostate SBRT patients and twenty selected lung SBRT patients. The selection criteria for the 20 lung patients were motivated by being able to construct a data set representative of common treatment tracking methods and dose prescriptions. To evaluate the impact of InTempo imaging, treatment plans were re‐optimized using the same optimization parameters and machine settings, except for the path set with the maximum number of nodes. To ensure a fair comparison, the study plans were prescribed using identical planning target volume coverage as the clinical treatment plans. Statistical analyses were performed using mean and standard deviation, dose metric plots, and a two‐sided Wilcoxon signed rank test with multiple testing correction to compare dose metrics between different path sets.

**Results:**

No statistically significant differences were observed among the Prostate, Prostate_Short, and their corresponding InTempo path sets in at least 8 of the 14 evaluated plan metrics, including prostate clinical tumor volume (CTV) V40Gy(%), conformity index, and homogeneity index. For example, the mean prostate CTV V40Gy (%) for the Prostate, Prostate_Short, and their corresponding InTempo path sets was 90.8 ± 4.7, 89.4 ± 4.7, 90.2 ± 3.9, 91.0 ± 7.0, respectively. However, compared with the Prostate path set, the Prostate_InTempo path set exhibited a statistically significant reduction in delivery time (*p* = 0.0010), number of beams, and bladder V18Gy (%), along with a statistically significant increase in the number of imaging beams (*p* = 0.0010). Additionally, Prostate_Short demonstrated statistically significant reductions in delivery time and number of beams compared with the Prostate path set, while the number of imaging beams remained statistically equivalent. In contrast, the Reduced_Prostate and Reduced_Prostate_InTempo sets consistently resulted in inferior dosimetric outcomes, with several plans deemed unoptimizable due to insufficient node availability.

For lung SBRT, statistically significant differences were observed in delivery time and the number of imaging beams between plans with and without InTempo. However, no statistical differences were found in dose distribution metrics between these two lung groups.

**Conclusions:**

InTempo‐compatible path sets do not significantly compromise plan quality for prostate or lung SBRT, provided adequate node availability. Specifically, the Prostate_InTempo and Prostate_Short path sets demonstrated a reduction in delivery time and an increase in adaptive imaging frequency compared with the Prostate path set. However, the Reduced_Prostate and Reduced_Prostate_InTempo result in inferior plan quality and reduced deliverability and should be used with caution. These findings support the selective use of InTempo imaging in SBRT planning without sacrificing dosimetric integrity.

## 1 INTRODUCTION

The CyberKnife® system (Accuray, Inc., Sunnyvale, CA) is a state of the art platform for stereotactic body radiotherapy (SBRT), designed to achieve submillimeter accuracy in radiation delivery. It features a compact 6 MV linear accelerator (linac) mounted on a Kuka robotic arm, allowing for highly flexible and precise beam delivery. One of the key advantages of the CyberKnife system is its real time image‐guided tracking, which ensures accurate tumor targeting by compensating for patient and organ motion. This real time tracking is facilitated by a stereo imaging system comprising ceiling‐mounted kV *X*‐ray sources and floor‐mounted detector panels. *X*‐ray images are acquired at user‐defined intervals before and during treatment, enabling continuous localization of the tumor. The three‐dimensional (3D) tumor position can be determined using either implanted fiducial markers or anatomical landmarks. The latest CyberKnife S7 system integrates an advanced multi‐leaf collimator (MLC) capable of delivering radiation at a dose rate of up to 1000 monitor units per minute (MU/min). This MLC enhancement has been shown to improve treatment efficiency while maintaining high dosimetric accuracy and plan quality.

The InTempo adaptive imaging system is an advanced component of the CyberKnife system designed to dynamically adjust imaging frequency based on tumor intrafractional motion. This InTempo feature enhances tumor tracking accuracy while minimizing unnecessary imaging, thereby optimizing treatment delivery.

The prostate is one of the primary clinical sites where the InTempo system is utilized, given that prostate motion can exhibit erratic displacements of up to 12 mm within an 8 min treatment session^1^. Studies have demonstrated that the InTempo system effectively adapts CyberKnife's imaging frequency in response to intrafractional prostate motion, ensuring high treatment precision throughout the procedure.^2^, ^3^, ^4^, ^5^

However, a notable limitation of the InTempo adaptive imaging system is the reduction in the number of available nodes for the treatment planning system (TPS). In CyberKnife, the nodes are the specific points where the robotic arm stops and delivers radiation beams. This node reduction occurs due to the exclusion of blocked imaging nodes, which are essential for TPS optimization. Table 1 presents the number of nodes corresponding to various prostate motion paths and their respective InTempo paths available for TPS initialization. Theoretically, the decrease in available nodes may compromise treatment plan quality, as InTempo‐based plans may demonstrate inferior dosimetric outcomes compared to standard plans incorporating a full set of nodes.

**TABLE 1:** Node numbers of different prostate path sets with MLC modality (Accuray precision version 3.3.1.3 [2]).

	Prostate	Prostate_ InTempo	Prostate_ short	Prostate_ InTempo_ short	Reduced_ prostate	Reduced_ prostate_ InTempo
MLC	91	64	69	50	24	23

The impact of a reduced number of nodes on the quality of InTempo‐based treatment plans has not previously been evaluated. Assessing the impact of the reduction of available nodes is crucial for CyberKnife users to determine whether the adoption of InTempo imaging is justified despite the potential limitations in treatment plan quality. In this study, we conducted a retrospective plan dosimetric comparison with and without InTempo imaging while maintaining consistent optimization settings.

## 2 METHODS

### 2.1 Ethical approval and patient selection

This retrospective study was reviewed and approved by the Institutional Review Board. The study included 12 prostate SBRT patients and 20 lung SBRT patients treated using our CyberKnife Precision S7 system. The prostate patient treatments were delivered between October 2023 and November 2025; only 12 prostate patients met our selection criteria during this period. The lung patient treatments were conducted between October 2023 and December 2024. The selection criteria for the 20 lung patients were motivated by being able to construct a data set representative of different tracking methods and dose prescriptions, which are detailed in the section of Lung SBRT Planning.

The cohort of prostate cancer patients was selected, as they represent the primary candidates for InTempo imaging due to significant intrafractional motion. Additionally, given the anatomical complexity of the prostate, with the bladder and rectum in proximity, we included the cohort of lung cancer patients to evaluate the effects of InTempo imaging in a different anatomical context. This approach allows for a more comprehensive assessment of how the InTempo imaging path influences overall treatment plan quality across multiple disease sites.

### 2.2 Prostate SBRT planning

Prostate SBRT patients were treated with a prescription dose of 36.25 Gy in five fractions, with a simultaneously boosted (SIB) dose of 40 Gy to the clinical target volume (CTV). Additionally, six of the twelve prostate patients received a further 44 Gy boost to the prostatic nodule. Fiducial tracking was chosen using four implanted gold seeds per patient. Each patient was also implanted with Barrigel to minimize the side effects to the rectum.

For target delineation, MRI T2‐weighted imaging was fused with planning CT to contour the prostate CTV, prostate lymph nodule, and urethra. Treatment plans were optimized using MLC and the Volo optimization algorithm, while dose calculations were performed with the Finite Size Pencil Beam (FSPB) algorithm using high resolution and lateral scaling.

Dose constraints were primarily based on NRG‐GU010^6^, RTOG 0938,^7^, ^8^ and our institutional protocols. Dose variation is the dose level outside the formal constraint but still allowed by the protocols. To control dose spillage, three to four auto‐shells were added around the planning target volume (PTV) at distances ranging from 5 mm to 50 mm. The urethra dose constraint was set to a maximum dose of ≤38.78 Gy with a variation of 43.5 Gy, while target goals included coverage of the PTV, CTV, and prostatic nodule. Exit beams were usually chosen for the testis and lower extremities to minimize unnecessary doses.

To evaluate the impact of different path sets on plan quality, all clinically treated plans were re‐optimized under identical optimization parameters and machine settings, except for the path set selection with the maximum number of nodes in the optimization setup. After one round of optimization, final dose calculations were performed at high resolution. To ensure an appropriate comparison, plans were prescribed using the same PTV coverage as the originally approved treatment plans. This process was applied to all the path sets, including Prostate, Prostate_Short, Reduced_Prostate, Prostate_InTempo, Prostate_InTempo_Short, and Reduced_Prostate_InTempo. The dosimetry metrics for plan comparison included conformity index (CI), homogeneity index (HI), delivery time (min), number of beams, number of imaging beams, total monitor units (MU), rectum maximum dose D0.03cc(cGy), rectum V29Gy(%), rectum V18Gy(%), bladder D0.03cc(cGy), bladder V37Gy(cc), bladder V18Gy(%), urethra D0.03cc(cGy), and prostate CTV V40Gy(%). Note the two types of beams: the 6MV FFF treatment beams from the Linac, which are used for delivering radiation, and the kV *X*‐ray imaging beams, which are used for adaptive imaging to track tumor motion and adjust for patient shifts. The definitions of CI and HI are:

$$\mathrm{CI}=\frac{\text{Total tissue volume that receives the prescription dose}}{\text{PTV volume that receives the prescription dose}}$$

$$\mathrm{HI}=\frac{\text{Plan maximum dose}}{\text{Prescription dose}}$$

### 2.3 Lung SBRT planning

Among the 20 lung SBRT patients, 11 were treated using fiducial with respiratory synchrony tracking, while 9 underwent lung with respiratory (non‐fiducial) tracking. The prescribed doses were 55 Gy in 5 fractions (12 patients), 50 Gy in 5 fractions (2 patients), and 54 Gy in 3 fractions (6 patients).

Treatment planning was performed using MLC‐based Volo optimization, and calculations were conducted using the Monte Carlo (MC) algorithm with high resolution (1% uncertainty). The full path (with fiducial with respiratory tracking or lung with respiratory tracking) was used in all clinical treatment plans. If a patient's arms were positioned at the same axial level as the tumor, only exit beams were permitted through the arms to minimize dose exposure to upper extremity structures.

For comparative analysis, 20 study plans were generated under identical planning settings after switching to the InTempo Imaging. The InTempo path set and fiducial synchrony method were used in the study plans after switching from plans that used the full path and Fiducial with Respiratory synchrony method. Accordingly, the InTempo path set and Spine Supine synchrony method were utilized in the study plans, replacing the Full path and Lung with Respiratory synchrony method. These changes were made because InTempo imaging is not currently integrated with synchrony respiratory motion tracking. The final dose was prescribed to maintain identical PTV coverage, and the following dosimetric parameters were evaluated: CI, R50 (R50% in RTOG0813^9^), HI, delivery time (min), number of beams, number of imaging beams, total MU, lung V20Gy (%), lung 12.5 Gy (cc) for patients with a prescription of 55 Gy/50 Gy in 5 fractions, and V12.4 Gy (cc) for patients with 54 Gy in 3 fractions.

### 2.4 Statistical analysis

For plan quality assessment, mean and standard deviation values were computed, and dose metrics were assessed graphically. A two‐sided Wilcoxon signed rank test was conducted using MATLAB version R2024a (MathWorks)^10^ to compare differences in dosimetric parameters. The Wilcoxon signed rank test was selected for our paired nonparametric data with a small sample size. Bonferroni correction for multiple testing was applied to the conventional significance level (*α* = 0.05). For example, in the pairwise comparison of the Prostate and Prostate_InTempo path sets in 12 patients, a *p*‐value < 0.0036 (= 0.05/14), rather than the conventional level of 0.05, was considered statistically significant, reflecting adjustment for 14 comparisons corresponding to 14 distinct plan metrics. Statistically marginal differences were further evaluated using the Holm–Bonferroni method.

## 3 RESULTS

### 3.1 Prostate SBRT plans

Table 2 presents the mean values of the 14 plan metrics for each path set. The means of the Prostate, Prostate_Short, Prostate_InTempo, and Prostate_InTempo_Short path sets were derived from the 12 prostate patients, while the means of Reduced_Prostate were from 11 patients, and those of Reduced_Prostate_InTempo were from nine patients. Patients 3 (two plans), 6 (one plan), and 12 (one plan) were unable to generate plans using the Reduced_Prostate and Reduced_Prostate_InTempo path sets. The average CI of the Prostate, Prostate_Short, Prostate_InTempo, Prostate_InTempo_Short path sets were 1.09 ± 0.04, 1.09 ± 0.04, 1.10 ± 0.04, and 1.14 ± 0.09, respectively. In contrast, the average CI of the plans using Reduced_Prostate and Reduced_Prostate_InTempo exceeded the plan tolerance of CI ≤ 1.20. The average delivery time (in minutes) of the Prostate, Prostate_Short, and their corresponding InTempo path sets were 21 ± 1, 20 ± 1, 19 ± 1, and 18 ± 1, respectively. An equal number of treatment beams and imaging beams were shown in plans with InTempo path sets. The average imaging beams for the Prostate, Prostate_Short, and their corresponding InTempo path sets were 28 ± 5, 24 ± 5, 34 ± 5, and 29 ± 3, respectively. The average prostate CTV V40Gy (%) for the Prostate, Prostate_Short, and their InTempo counterparts were 90.8 ± 4.7, 89.4 ± 4.7, 90.2 ± 3.9, and 91.0 ± 7.0, respectively. The means of the dose metrics for the rectum, bladder, and urethra in Table 2 were acceptable and within plan dose constraints and variations.

**TABLE 2:** Means of the plan metrics of each prostate path set.

Metrics	Prostate	Prostate_ InTempo	Prostate_ short	Prostate_ InTempo_ short	Reduced_ prostate	Reduced_ prostate_ InTempo
CI	1.09 ± 0.04	1.10 ± 0.04	1.09 ± 0.04	1.14 ± 0.09	2.34 ± 1.63	1.83 ± 0.87
HI	1.26 ± 0.06	1.26 ± 0.05	1.26 ± 0.06	1.28 ± 0.06	1.62 ± 0.46	1.49 ± 0.28
Total MU	13888.3 ± 1514.6	15230.0 ± 1295.7	12776.8 ± 3636.0	14776.9 ± 1322.7	12364.9 ± 2337.4	12244.2 ± 2098.1
Delivery time (min)	21 ± 1	19 ± 1	20 ± 1	18 ± 1	14 ± 3	15 ± 2
# of Beams	44 ± 6	34 ± 5	38 ± 5	29 ± 3	16 ± 7	17 ± 6
# of Imaging beams	28 ± 5	34 ± 5	24 ± 5	29 ± 3	15 ± 7	17 ± 6
Rectum D0.03cc (cGy)	3716 ± 185	3717 ± 243	3711 ± 190	3791 ± 165	4010 ± 377	3914 ± 275
Rectum V29Gy (%)	4.4 ± 2.9	3.7 ± 2.2	4.2 ± 2.4	4.1 ± 2.4	12.0 ± 7.6	11.9 ± 10.1
Rectum V18Gy (%)	18.6 ± 5.3	17.2 ± 5.0	18.4 ± 4.7	19.8 ± 5.4	32.6 ± 8.2	35.0 ± 9.3
Bladder D0.03cc (cGy)	3973 ± 74	3995 ± 65	3981 ± 80	4093 ± 197	5104 ± 749	5006 ± 808
Bladder V37Gy (cc)	2.87 ± 0.99	2.98 ± 0.98	2.83 ± 0.98	3.44 ± 1.26	12.92 ± 8.79	13.26 ± 12.80
Bladder V18Gy (%)	23.7 ± 10.2	20.7 ± 8.1	23.3 ± 9.8	23.6 ± 10.3	31.7 ± 19.4	28.6 ± 18.7
Urethra D0.03cc (cGy)	3970 ± 100	4006 ± 107	3970 ± 105	4102 ± 193	4667 ± 420	4579 ± 361

Table 3 lists the *p*‐values of the plan metrics for pairwise comparison of different prostate path sets. Only six of fourteen metrics with statistically significant differences were listed. A *p*‐value < 0.0036 (= 0.05/14) was considered statistically significant. Compared with the Prostate path set, Prostate_InTempo demonstrated statistically significantly shorter delivery times, a reduced number of treatment beams, an increased number of imaging beams, and lower bladder V18Gy (%). When compared with Prostate_Short, Prostate_InTempo_Short exhibited a reduced number of treatment beams, as well as higher maximum doses D0.03cc(cGy) to the bladder and urethra. No statistically significant differences were found in the following dosimetric endpoints across the Prostate, Prostate_Short, and their InTempo equivalents: CI, HI, total MU, rectum maximum dose of D0.03cc (cGy), rectum V29Gy (%), rectum V18Gy (%), bladder V37Gy (cc), and prostate CTV V40Gy (%). Additionally, the Reduced_Prostate and Reduced_Prostate_InTempo groups did not exhibit any statistically significant difference. Furthermore, Prostate_Short showed a statistically significantly shorter delivery time and fewer treatment beams compared with the Prostate path set. Finally, the plan with Prostate_InTempo_Short demonstrated a statistically significantly higher bladder maximum dose D0.03cc(cGy) than those with Prostate_InTempo.

**TABLE 3:** *p* Values for pairwise comparison of prostate different path sets. Only plan metrics with statistically significant differences were listed. A *p* value < 0.0036 was considered significant accounting for the Bonferroni correction.

Metrics	Prostate vs Prostate_ InTempo	Prostate_ short vs Prostate_ InTempo_short	Reduced_ prostate vs reduced_ prostate_ InTempo	Prostate vs prostate_ short	Prostate_ InTempo vs prostate_ InTempo_short
Delivery time(min)	0.0010	0.0059	0.7500	0.0010	0.0625
Number of beams	0.0005	0.0010	0.0938	0.0034	0.0039
Number of imaging beams	0.0010	0.0098	0.7500	0.0166	0.0039
Bladder D0.03cc (cGy)	0.0278	0.0024	0.1289	1.0000	0.0005
Bladder V18Gy (%)	0.0005	0.7197	0.1367	0.0737	0.1299
Urethra D0.03cc (cGy)	0.0366	0.0024	1.0000	0.9253	0.0088

**FIGURE 1:**
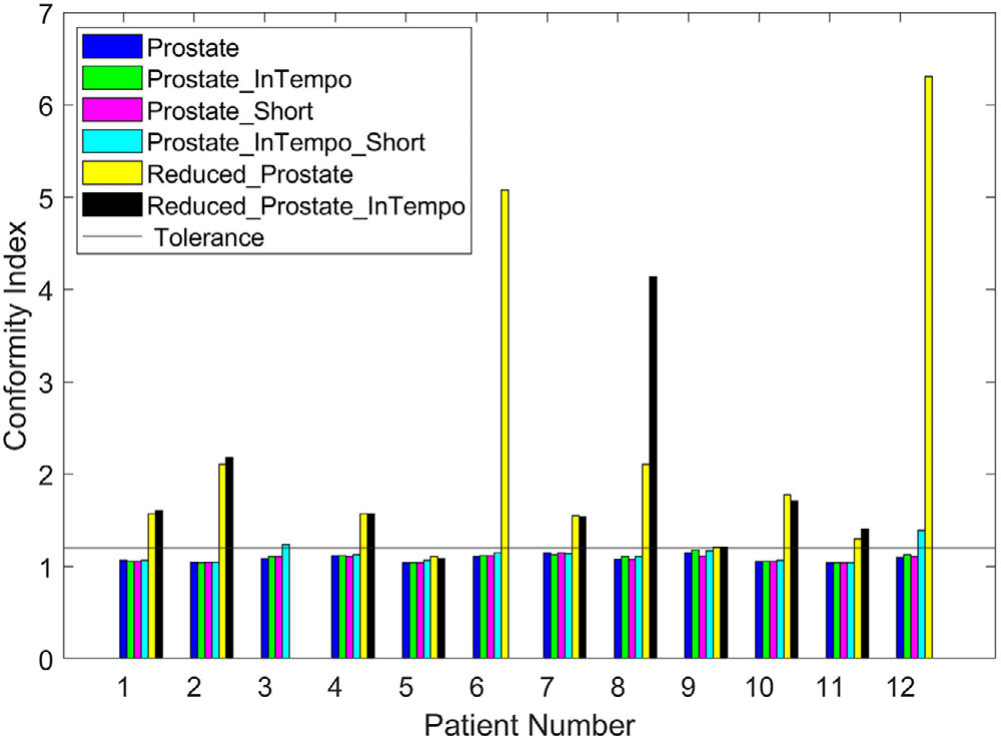
CI values for the twelve prostate SBRT study plans across six path set configurations. The CI tolerance threshold of 1.2 is indicated by the horizontal black line.

Figure 1 illustrates the CI values for the twelve prostate SBRT cases across all evaluated path sets. Missing CI values were reported for patients 3 (two plans), 6 (one plan), and 12 (one plan). The CI tolerance threshold of 1.2 is denoted by a horizontal black line. Notably, the plans using the Prostate_InTempo_Short path for patients 3 and 12 exceeded this threshold, indicating suboptimal conformity. Moreover, ten of twelve patients had CI values above the tolerance when planned with the Reduced_Prostate or Reduced_Prostate_InTempo paths. In contrast, CI values across the remaining path sets were consistently within tolerance and visually similar.

**FIGURE 2:**
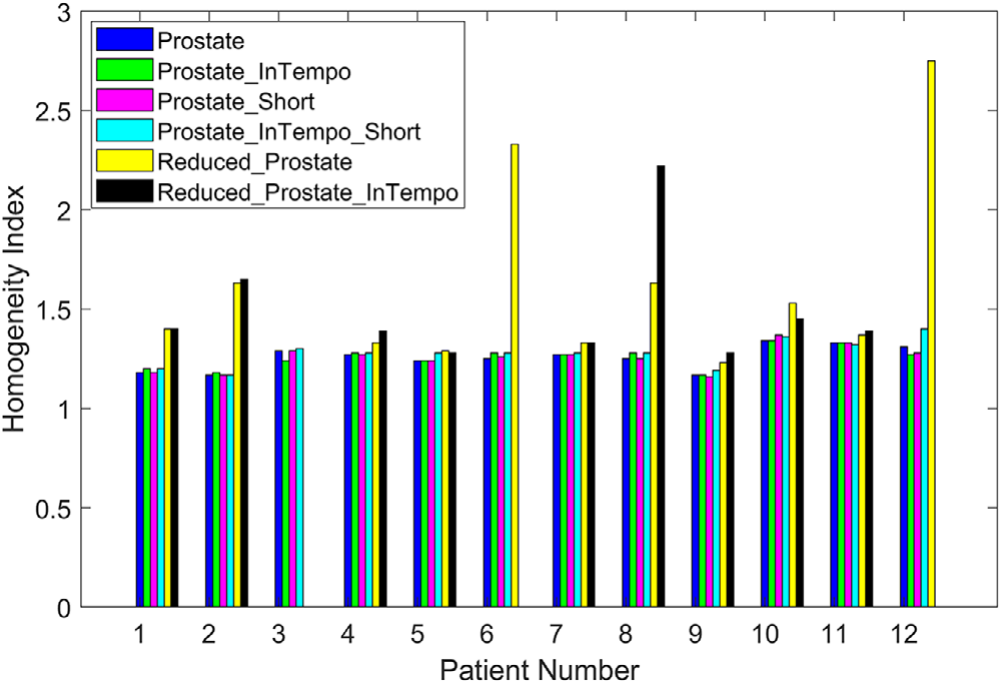
HI values for the twelve prostate SBRT study plans across six path sets.

Figure 2 presents the HI values for all paths. Plans utilizing the Reduced_Prostate and Reduced_Prostate_InTempo paths generally demonstrated higher HIs than those using standard and InTempo‐compatible Prostate or Prostate_Short configurations, indicating inferior dose uniformity. At our clinic, based on the references,^7^, ^8^, ^11^, ^12^ the HI ≤ 1.24 and HI ≤ 1.34 are preferred in the treatment plans for the prostate PTV 36.25 Gy/CTV 40 Gy SIB and PTV 36.25 Gy/CTV 40 Gy/Nodule 44 Gy SIB, respectively.

**FIGURE 3:**
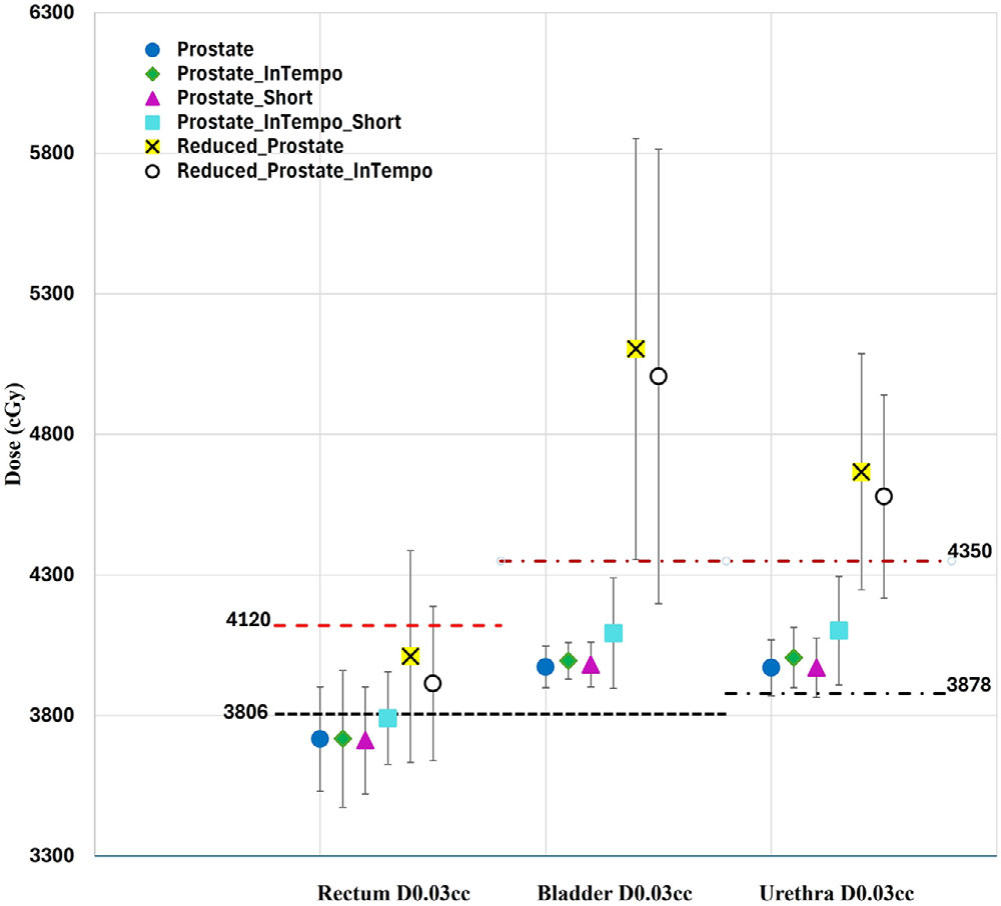
Average maximum doses to the rectum, bladder, and urethra across the prostate SBRT plans using six path sets. Horizontal lines represent protocol dose constraints and variation thresholds.

Figure 3 displays the average maximum doses delivered to the rectum, bladder, and urethra across different path sets. Reference dose constraints and variation thresholds are overlaid: 3806 cGy (constraint) and 4120 cGy (variation) for the rectum, 3806 cGy and 4350 cGy for the bladder, and 3878 cGy and 4350 cGy for the urethra. These reference lines provide a benchmark for evaluating organ‐at‐risk (OAR) sparing across planning techniques. Due to the presence of Barrigel implants, rectal doses remained within safe thresholds across all paths. The bladder and urethra maximum doses remained within acceptable limits for plans using Prostate, Prostate_Short, and their corresponding InTempo paths. However, the Reduced_Prostate and Reduced_Prostate_InTempo paths exceeded the defined dose constraints and variations for both organs, indicating elevated toxicity risks.

**FIGURE 4:**
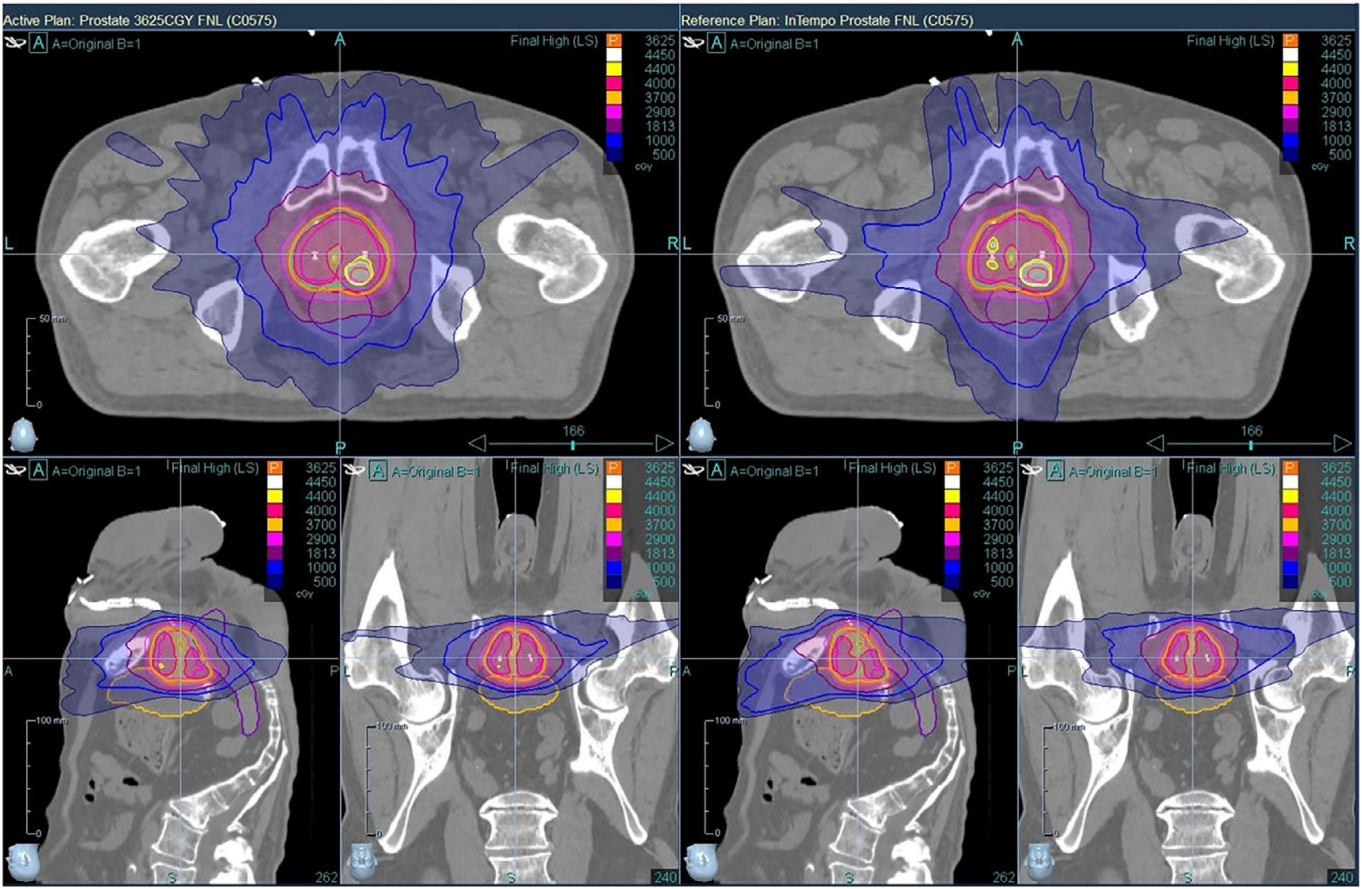
Comparison of dose distributions between the Prostate path plan (left) and Prostate_InTempo path plan (right) of patient No.7. Both plans demonstrate high‐quality, conformal dose coverage.

**FIGURE 5:**
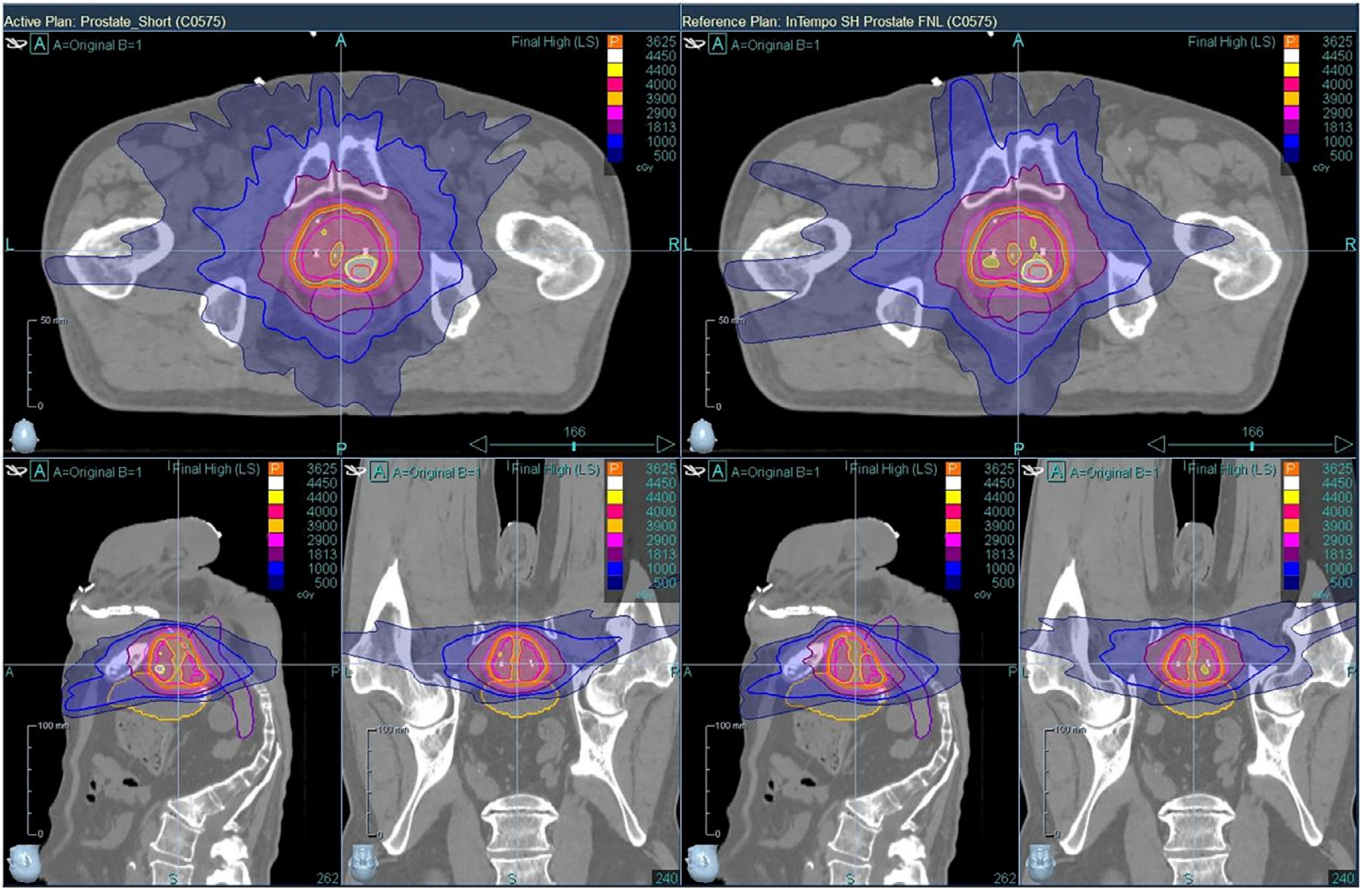
Comparison of dose distributions between the Prostate_Short path (left) and the Prostate_InTempo_Short path (right) of patient No.7. The axial views show small differences in the dose distribution.

**FIGURE 6:**
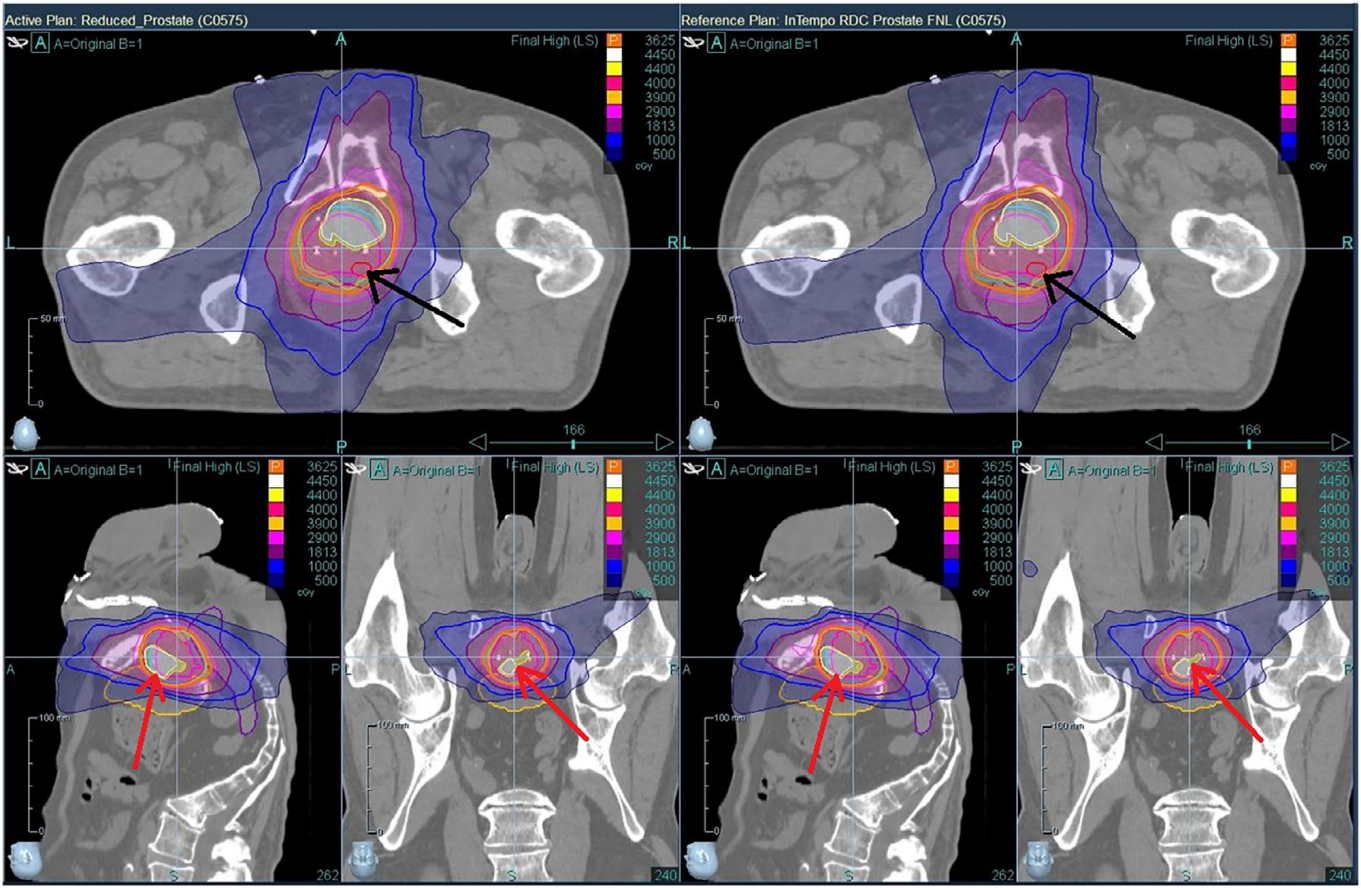
Comparison of dose distributions between the Reduced_Prostate path (left) and the Reduced_Prostate_InTempo path (right) of patient No.7. Both plans demonstrate similar, yet suboptimal dosimetric performance, with excessive dose exposure to the bladder and urethra surpassing clinical acceptability thresholds (indicated by the red arrows). In this case, the prostate nodule is underdosed (as shown by the black arrows).

Figures 4 through 6 provide visual comparisons of dose distributions using color‐wash overlays. Figures 4 and 5 compare the Prostate versus Prostate_InTempo and Prostate_Short versus. Prostate_InTempo_Short plans, respectively. In both comparisons, dose coverage was conformal with no major visible differences between the InTempo and non‐InTempo plans. Figure 6, in contrast, shows the dose distributions for the Reduced_Prostate and Reduced_Prostate_InTempo paths, both of which reveal poor conformity and noticeable overdosage to the bladder and urethra—consistent with the quantitative findings.

### 3.2 Lung SBRT plans

Table 4 shows the 9 metric characteristics for the lung SBRT plans with and without InTempo imaging for the cohort of 20 lung patients. The *p*‐values were calculated with the Wilcoxon signed rank test. A *p*‐value < 0.0056 (= 0.05/9) was considered significant, accounting for the Bonferroni correction. A statistically significant difference in average delivery time was observed: 35 ± 2 min for plans without InTempo imaging versus 23 ± 3 min for those with InTempo (*p* < 0.00010). Similarly, the number of imaging beams differed significantly between the two groups (p < 0.00010). In addition, the CI revealed a statistical difference between the full path and InTempo. However, no statistically significant differences were found in key dosimetric parameters, including R50, HI, total MU, Lung V20Gy (%), and Lung V12.5 Gy (cc) or V12.4 Gy (cc) for plans prescribed over three fractions.

**TABLE 4:** Mean values and *p* values of the plan metrics for the lung different path sets. A *p* value < 0.0056 was considered significant, accounting for the Bonferroni correction.

Metrics	Mean of option		*p* Value of full path vs InTempo
	Full path	InTempo	
CI	1.10 ± 0.05	1.12 ± 0.05	0.002
R50	4.23 ± 0.77	4.33 ± 0.79	0.0123
HI	1.35 ± 0.06	1.37 ± 0.08	0.1299
Delivery time (min)	35 ± 2	23 ± 3	< 0.00010
# of Beams	42 ± 5	38 ± 6	0.0142
# of Imaging beams	26 ± 5	38 ± 6	< 0.00010
Total MU	17631.1 ± 3161.2	18697.7 ± 3309.0	0.008
Lungs V20Gy (%)	3.4 ± 2.0	3.4 ± 2.0	0.1196
^*^Lungs V12.5 Gy (cc)	202.7 ± 117.8	214.7 ± 123.7	0.01

* Lungs V12.4 Gy(cc) for plans with the prescription of 54 Gy in 3 fractions.

Table 5 includes the means and *p*‐values of the plan metrics for the subgroups (11 lung patients with fiducials and 9 patients without fiducials). A statistically significant reduction in delivery time and a statistically significant increase in the number of imaging beams were presented in both subgroups when comparing the plans with and without InTempo. In the subgroup without fiducials, no other dose metrics demonstrated statistical differences. However, the subgroup with fiducials also showed a statistical difference in total MU between the full path and InTempo. No statistically significant differences were observed in R50, HI, number of beams, lungs V20Gy (%), or lungs V12.5 Gy (cc)/V12.4 Gy (cc) between the Full path and InTempo plans in either subgroup.

**TABLE 5:** Means and *p* values of the plan metrics for the subgroups (lung with and without fiducials). A *p* value < 0.0056 was considered significant.

Metrics	Lung with fiducials			Lung without fiducials		
	Full path	InTempo	p‐value	Full path	InTempo	*p* Value
CI	1.10 ± 0.06	1.12 ± 0.06	0.0117	1.10 ± 0.04	1.12 ± 0.05	0.0547
R50	4.49 ± 0.89	4.59 ± 0.88	0.0674	3.92 ± 0.46	4.02 ± 0.58	0.1523
HI	1.34 ± 0.07	1.36 ± 0.07	0.0781	1.35 ± 0.06	1.38 ± 0.09	0.418
Delivery time (min)	35 ± 3	21 ± 2	< 0.0010	33 ± 1	25 ± 3	0.0039
Number of beams	39 ± 5	36 ± 6	0.1084	45 ± 4	40 ± 5	0.0977
Number of imaging beams	25 ± 5	36 ± 6	< 0.0010	28 ± 4	40 ± 5	0.0039
Total MU	16493.3 ± 2909.2	18111.5 ± 2721.7	< 0.0010	19021.9 ± 3032.9	19414.0 ± 3961.8	0.4961
Lungs V20Gy (%)	2.3 ± 0.9	2.3 ± 1.0	0.1875	4.8 ± 2.2	4.8 ± 2.1	0.4531
^*^Lungs V12.5 Gy (cc)	146.8 ± 71.8	156.6 ± 86.4	0.083	271.0 ± 130.3	285.7 ± 128.9	0.0547

* Lungs V12.4 Gy(cc) for plans with the prescription of 54 Gy in 3 fractions.

**FIGURE 7:**
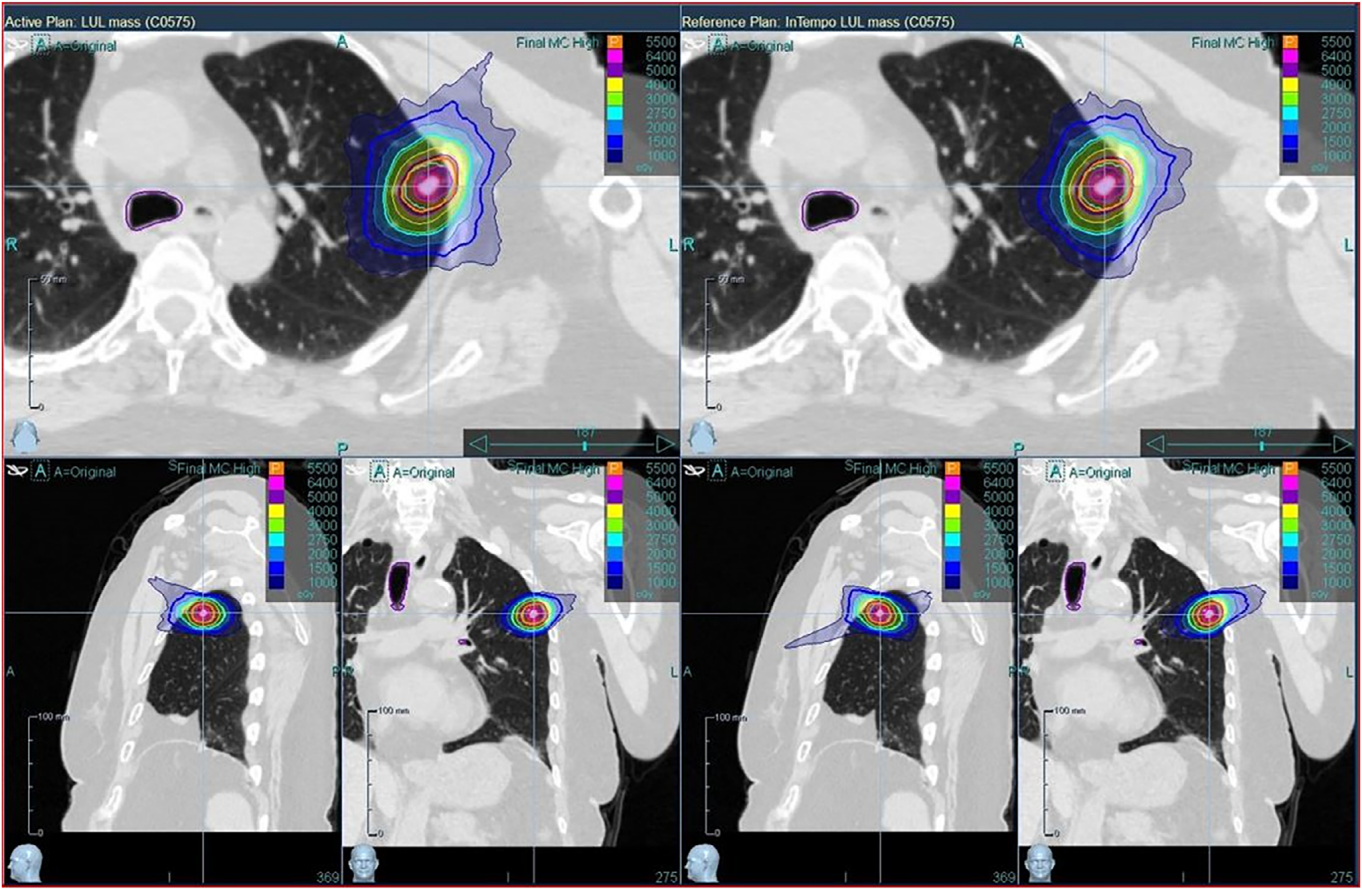
Lung SBRT plan dose comparison between the full path (left) and InTempo path set (right). Small distribution differences were shown on the 3D views.

Figure 7 illustrates representative dose distributions for lung SBRT plans using the Full path without InTempo imaging (left) and the InTempo imaging path (right). Both plans demonstrate excellent target conformity and clinical acceptability, with no visible compromise in plan quality attributable to the use of InTempo imaging.

## 4 DISCUSSION

This study systematically evaluated the impact of the InTempo path on CyberKnife SBRT plan quality for prostate and lung cancers. Notably, plans with the InTempo path set include an equal number of treatment beams and imaging beams. Therefore, prior to the delivery of each treatment beam, a corresponding set of kV *X*‐ray images is acquired to verify the tumor position or tracking volume. This pre‐beam verification enhances the accuracy of dose delivery to the target when using InTempo, compared with dose delivery in a standard non‐InTempo path set where *X*‐ray imaging is not performed before each treatment beam. This represents a key advantage of InTempo adaptive imaging in CyberKnife treatments.

The findings from Table 2 and Figures 1, 2, 3, 4, 5, 6 demonstrated that Prostate, Prostate_Short, Prostate_InTempo, and Prostate_InTempo_Short path sets produced comparable high‐quality prostate SBRT plans. No statistically significant differences were observed among these path sets in prostate CTV V40Gy(%), CI, HI, total MU, rectum D0.03cc (cGy), rectum V29Gy (%), rectum V18Gy (%), or bladder V37Gy (cc), with all these metrics within the plan dose constraints and variations. In addition, plans using the Prostate_InTempo exhibited a statistically significant reduction in delivery time, a decrease in the number of beams, an increase in the number of imaging beams, and a lower bladder V18Gy (%) compared with those of the Prostate path set. Furthermore, Prostate_Short path demonstrated a statistically significant reduction in delivery time and number of treatment beams when compared with Prostate path, while the number of imaging beams remained statistically equivalent.

Our results showed that reducing the maximum nodes in the plan optimization leads to a statistically significant reduction in delivery time. These findings are consistent with previous work by Van de Water et al. ^13^, who reported that node reduction can substantially decrease treatment time with minimal compromise in dosimetric quality. This outcome is expected, as a lower number of nodes requires fewer robotic repositionings, thereby reducing the overall beam‐on and delivery time. Based on these results, one planning strategy to improve prostate SBRT treatment efficiency without dosimetric loss is to use a moderate maximum node number in the range of 64 to 69.

A larger number of imaging beams (Prostate_InTempo) or an equivalent number of imaging beams (Prostate_Short) delivered within a shorter treatment time enables more frequent adaptive imaging. Prostate_InTempo and Prostate_Short path sets demonstrated clinically meaningful advantages by achieving reduced delivery time and increased imaging frequency while maintaining high plan quality. Shorter delivery times may improve patient comfort and reduce dose uncertainty associated with intrafraction motion. Additionally, more frequent adaptive imaging may enhance dose delivery accuracy and PTV coverage, as previously reported by Rose et al.^14^ and Van De Water et al.^15^ Regarding the imaging dose, it is important to note that kV portal images increase the patient dose by only 1–3 mGy per image. The additional *X*‐ray imaging beams are anticipated to contribute minimally to the overall patient dose when compared to the therapeutic dose.

Although the Prostate, Prostate_Short and their InTempo counterparts generated similar high‐quality prostate SBRT plans, further analyses of CI values and maximum dose thresholds uncovered limitations in the Reduced_Prostate and Reduced_Prostate_InTempo configurations. Specifically, 83% (10 of 12) of these plans exceeded the CI tolerance, and average maximum doses to the bladder and urethra either exceeded variation thresholds or were not obtainable due to plan generation failure. A Review of the failed plans for patients 3, 6, and 12 revealed that tissue boundary sampling had been employed in the original plan optimization, which may require more nodes in the optimization and possibly contribute to the inability to create Reduced_Prostate‐based study plans due to insufficient node availability.

For lung SBRT, as shown in Table 4, InTempo‐enabled plans demonstrated a notable reduction in delivery time (23 ± 3 min vs. 35 ± 2 min) and a marked increase in the number of imaging beams (38 ± 6 vs. 26 ± 5) compared with full path plans. Statistically significant differences in the CI were also observed between the InTempo and Full path plans. Based on the lung SBRT subgroup analysis with and without fiducials presented in Table 5, InTempo influenced the fiducial subgroup not only in terms of delivery time and number of imaging beams but also in total monitor units. Despite these differences, Tables 4 and 5 and Figure 7 demonstrated that lung SBRT plans with and without InTempo achieved excellent target conformity and acceptable dose distribution. This stability is likely attributable to the higher node counts available during plan optimization—typically 75–90 for clinical plans and 71 for study plans—which ensured sufficient beam coverage 42 ± 5 beams without InTempo vs. 38 ± 6 beams with InTempo. The node reduction can decrease treatment time with minimal compromise in dosimetric quality. However, the big difference in the lung estimated delivery time is from the estimated patient setup time. In our CyberKnife S7 system, the estimated patient setup time for the fiducial with respiratory or lung with respiratory tracking is 15 min; however, for the fiducial or spine supine tracking is 5 min.

It is important to note that InTempo imaging is not currently integrated with Synchrony Respiratory Motion Tracking. In clinical practice, when lung SBRT plans employing fiducial with respiratory or lung with respiratory tracking are converted to plans with fiducial or spine supine tracking, the clinical scenarios and corresponding PTVs should appropriately differ. However, the primary objective of this study is to evaluate the impact of the InTempo path on plan quality. The authors do not propose treating patients with identical PTVs after a change in tracking mode. One limitation of this study is the use of the same lung PTV for the comparison between InTempo and Full Path plans. The dosimetric comparison among different tracking methods may not be a completely analogous evaluation. In our clinical practice, fiducial with respiratory tracking and lung with respiratory tracking typically require three sets of kV images every 60 sec, whereas fiducial tracking and spine supine tracking generally require one set of kV images every 20 sec. Although these four tracking approaches employ similar adaptive imaging principles, important differences exist in target definition. For spine supine tracking, the internal target volume (ITV) is created from 4DCT images; however, for lung respiratory tracking, the ITV is contoured from breath‐hold CT (usually exhale CT). Therefore, even if the PTV is created with the same 5 mm expansion from the ITV, the PTV volume with spine tracking is usually larger than that with lung respiratory tracking. Without real motion, the planning system makes similar plans, which is a limitation of the planning study.

The second limitation of this study is the limited data size from just one institute. Future work will validate the present findings using a larger, independent data set. Both the Wilcoxon signed‐rank test and Bonferroni correction are influenced by sample size. For example, the CI demonstrated a statistically significant difference between the full path and InTempo in the cohort of 20 lung patients. However, when the cohort was divided into subgroups with fiducials (11 patients) and without fiducials (9 patients), no statistically significant differences in the CI were observed between these path sets. Finally, the differences in dose distributions, metric doses, and the estimated delivery times in this study were derived from the TPS. Future work will investigate the differences in both dose metrics and projection accuracy with real delivery data.

## 5 CONCLUSIONS

For prostate SBRT, the Prostate, Prostate_Short, Prostate_InTempo, and Prostate_InTempo_Short path sets exhibited comparable and high‐quality dosimetry. Specifically, the plans with the Prostate_InTempo and Prostate_Short path sets demonstrated a reduction in delivery time and an increase in adaptive imaging frequency relative to those with the Prostate path set. However, the Reduced_Prostate and Reduced_Prostate_InTempo path sets consistently yielded inferior dosimetric outcomes and, in several cases, failed to generate clinically acceptable plans due to node limitations. It is therefore recommended to use these path sets with fewer than ∼50 nodes cautiously.

In lung SBRT, treatment plans created with the full path and those incorporating InTempo imaging showed acceptable dosimetric quality, despite the presence of statistically significant differences in delivery time and the number of imaging beams. Currently, InTempo cannot be used in the lung SBRT with the fiducial or lung respiratory tracking; however, the node‐reduction technique is helpful to shorten the lung treatment.

This study was based on the TPS, and our results should be confirmed with larger patient data and real treatment delivery.

## AUTHOR CONTRIBUTIONS

Yingcui Jia and Qianyi Xu contributed to the study conception, design, and analysis. All authors contributed to the clinical data acquisition of this study. Yingcui Jia wrote the first draft of the manuscript, and the rest of the authors commented and edited the manuscript. We confirm that all coauthors contributed to the study.

## CONFLICT OF INTEREST STATEMENT

The authors declare no conflicts of interest.
